# Supplementary material for: Overcoming Current and Preventing Future Nosocomial Outbreaks during the COVID-19 Pandemic: Lessons Learned at Three Hospitals in Japan
Source: Int J Environ Res Public Health. 2021 Sep 28;18(19):10226. doi: 10.3390/ijerph181910226 (PMC8508432; doi:10.3390/ijerph181910226)
Supplement: Supplementary file 1 [file ijerph-18-10226-s001.zip › ijerph-1380151-supplementary.pdf]

Table S1. List of materials referred for three hospitals.

Hospital	No	Type of source	Date published	URL
A	1	MOHSW cluster team' report	15/4/2020	http://www.eijuhp.com/user/media/eiju/chousasiennhoukoku.pdf
A	2	Analysis by IASR	31/7/2020	https://www.niid.go.jp/niid/ja/typhi-m/iasr-reference/2523-related-articles/related-articles-485/9759-485r06.html
A	3	Staff report	8/6/2020	http://www.eijuhp.com/user/media/eiju/20200608saikainiatatte.pdf
A	4	Staff report	8/6/2020	https://s3-us-west-2.amazonaws.com/jnpc-prd-public-oregon/files/2020/06/2373da39-fbdb-4095-b7f1-811d3f997d2a.pdf
A	5	Staff report	8/6/2020	http://www.eijuhp.com/user/media/eiju_kenshin/layout/20200701siryou2.pdf
A	6	Web newspaper	1/7/2020	https://www.asahi.com/articles/ASN716CTWN71ULBJ00B.html?iref=pc_photo_gallery_bottom
B	7	Hospital announcement	24/3/2021	http://www.eijuhp.com/user/media/20210324kono1nenwofurikaerimashite..pdf
A	8	Hospital general information	22/8/2021	http://www.eijuhp.com/floor_guide.html
A	9	Announcement from Keio University hospital	1/5/2020	http://www.hosp.keio.ac.jp/st/management/info/covid-19_info_0501.pdf (Reported date: 21/4/2020, 6/4/2020, 27/3/2020, 26/3/2020）
B	10	Hospital general information	-	https://www.keiyukai-group.com/yoshi-hp/pamphlet/pdf/yoshidapamphlet.pdf#zoom=50
B	11	Hospital general information	-	https://www.keiyukai-group.com/yoshi-hp/about/organization/
B	12	Hospital report	20/1/2021	https://www.keiyukai-group.com/yoshi-hp/uploads/sites/2/2021/01/yoshida_cluster.pdf
C	13	Hospital report	26/2/2021	https://www.chuobyoin.or.jp/2021/02/covid19-report20210226/
C	14	Hospital report	29/2/2021	https://www.chuobyoin.or.jp/2021/01/covid19-report20210129/
C	15	Hospital report	15/1/2021	https://www.chuobyoin.or.jp/2021/01/notice_covid19/
C	16	Web newspaper	18/1/2021	https://www.tokyo-np.co.jp/article/80628
C	17	Web newspaper	21/1/2021	https://www.asahi.com/articles/ASP1N740NP1NUTNB00S.html
C	18	Hospital Announcement	28/11/2020	https://www.chuobyoin.or.jp/2020/11/covid-19_report20201128-01/
C	19	Hospital Announcement	2/12/2020	https://www.chuobyoin.or.jp/2020/12/covid-19_report20201128-01-2/
C	20	Hospital Announcement	4/12/2020	https://www.chuobyoin.or.jp/2020/12/covid-19_report20201128-01-2-2/
C	21	Hospital Announcement	10/12/2020	https://www.chuobyoin.or.jp/2020/12/covid-19_report20201210/
C	22	Hospital	19/12/202	https://www.chuobyoin.or.jp/2020/12/covid-

		Announcement	0	19_report20201210-2/
C	23	Hospital Announcement	21/12/2020	https://www.chuobyoin.or.jp/2020/12/covid-19_report20201221/
C	24	Hospital Announcement	23/12/2020	https://www.chuobyoin.or.jp/2020/12/covid-19_report20201223/
C	25	Hospital Announcement	29/12/2020	https://www.chuobyoin.or.jp/2020/12/covid-19_report20201223-2/
C	26	Hospital Announcement	5/1/2021	https://www.chuobyoin.or.jp/2021/01/covid-19_report20210105/
C	27	Hospital Announcement	8/1/2021	https://www.chuobyoin.or.jp/2021/01/covid-19_report20210108/
C	28	Hospital Announcement	19/1/2021	https://www.chuobyoin.or.jp/2021/01/covid-19-report20210119/
C	29	Hospital Announcement	20/1/2021	https://www.chuobyoin.or.jp/2021/01/covid-19-report20210120/
C	30	Hospital Announcement	21/1/2021	https://www.chuobyoin.or.jp/2021/01/covid-19-report20210121-2/
C	31	Hospital Announcement	22/1/2021	https://www.chuobyoin.or.jp/2021/01/covid-19-report20210122/
C	32	Hospital Announcement	23/1/2021	https://www.chuobyoin.or.jp/2021/01/covid-19-report20210123/
C	33	Hospital Announcement	25/1/2021	https://www.chuobyoin.or.jp/2021/01/covid-19-report20210125/
C	34	Hospital Announcement	26/1/2021	https://www.chuobyoin.or.jp/2021/01/covid-19-report20210126/
C	35	Hospital Announcement	27/1/2021	https://www.chuobyoin.or.jp/2021/01/covid-19-report20210127/
C	36	Hospital Announcement	28/1/2021	https://www.chuobyoin.or.jp/2021/01/covid-19-report20210128/
C	37	Hospital Announcement	29/1/2021	https://www.chuobyoin.or.jp/2021/01/covid-19-report20210128-2/
C	38	Hospital Announcement	29/1/2021	https://www.chuobyoin.or.jp/2021/01/covid19-report20210129/
C	39	Hospital Announcement	30/1/2021	https://www.chuobyoin.or.jp/2021/01/covid-19-report20210128-2-2/
C	40	Hospital Announcement	1/2/2021	https://www.chuobyoin.or.jp/2021/02/covid-19-report20210201/
C	41	Hospital Announcement	2/2/2021	https://www.chuobyoin.or.jp/2021/02/covid-19-report20210202/
C	42	Hospital Announcement	3/2/2021	https://www.chuobyoin.or.jp/2021/02/covid-19-report20210203/
C	43	Hospital Announcement	4/2/2021	https://www.chuobyoin.or.jp/2021/02/covid-19-report20210204/
C	44	Hospital Announcement	5/2/2021	https://www.chuobyoin.or.jp/2021/02/covid-19-report20210205/
C	45	Hospital Announcement	6/2/2021	https://www.chuobyoin.or.jp/2021/02/covid-19-report20210206/
C	46	Hospital Announcement	8/2/2021	https://www.chuobyoin.or.jp/2021/02/covid-19-report20210208/
C	47	Hospital Announcement	8/2/2021	https://www.chuobyoin.or.jp/2021/02/medical-care20210208/

C	48	Hospital Announcement	9/2/2021	https://www.chuobyoin.or.jp/2021/02/covid-19-report20210209/
C	49	Hospital Announcement	10/2/2021	https://www.chuobyoin.or.jp/2021/02/covid-19-report20210210/
C	50	Hospital Announcement	12/2/2021	https://www.chuobyoin.or.jp/2021/02/covid-19-report20210212/
C	51	Hospital Announcement	13/2/2021	https://www.chuobyoin.or.jp/2021/02/covid-19-report20210212-2/
C	52	Hospital Announcement	15/2/2021	https://www.chuobyoin.or.jp/2021/02/covid-19-report20210215/
C	53	Hospital Announcement	16/2/2021	https://www.chuobyoin.or.jp/2021/02/covid-19-report20210216/
C	54	Hospital Announcement	17/2/2021	https://www.chuobyoin.or.jp/2021/02/covid-19-report20210217/
C	55	Hospital Announcement	18/2/2021	https://www.chuobyoin.or.jp/2021/02/covid-19-report20210218/
C	56	Hospital Announcement	19/2/2021	https://www.chuobyoin.or.jp/2021/02/covid-19-report20210219/
C	57	Hospital Announcement	20/2/2021	https://www.chuobyoin.or.jp/2021/02/covid-19-report20210220/
C	58	Hospital Announcement	22/2/2021	https://www.chuobyoin.or.jp/2021/02/covid-19-report20210222/
C	59	Hospital Announcement	24/2/2021	https://www.chuobyoin.or.jp/2021/02/covid-19-report20210224/
C	60	Hospital Announcement	25/2/2021	https://www.chuobyoin.or.jp/2021/02/covid-19-report20210225/
C	61	Hospital Announcement	26/2/2021	https://www.chuobyoin.or.jp/2021/02/covid19-report20210226/
C	62	Hospital Announcement	1/3/2021	https://www.chuobyoin.or.jp/2021/03/infection-prevention20210311/
C	63	Hospital Announcement	-	https://www.chuobyoin.or.jp/hospitalization/visitor/
C	64	Hospital map	-	https://www.chuobyoin.or.jp/floormap/all/
C	65	Hospital general information	-	https://www.chuobyoin.or.jp/department/index/list/
